# Anomalous Papillary Muscle Insertion Causing Dynamic Left Ventricular Outflow Tract Obstruction without Hypertrophic Obstructive Cardiomyopathy

**DOI:** 10.1155/2017/9878049

**Published:** 2017-05-15

**Authors:** Ravi Korabathina, Katherine Chiu, Hugh M. van Gelder, Arthur Labovitz

**Affiliations:** ^1^Department of Cardiovascular Sciences, University of South Florida Morsani College of Medicine, Bayfront Health St. Petersburg, St. Petersburg, FL, USA; ^2^Department of Cardiology, Bayfront Health St. Petersburg, St. Petersburg, FL, USA; ^3^Division of Cardiovascular Surgery, Bayfront Health St. Petersburg, St. Petersburg, FL, USA; ^4^Department of Cardiovascular Sciences, University of South Florida Morsani College of Medicine, Tampa, FL, USA

## Abstract

Anomalous papillary muscle insertion directly into the surface of the mitral valve leaflet is rare, especially in a subject without apparent evidence of hypertrophic cardiomyopathy. We present a case of this isolated congenital malformation producing two hemodynamic sequelae of dynamic left ventricular outflow tract obstruction and severe mitral regurgitation.

## 1. Introduction

Congenital malformations of the papillary muscles, including their direct insertion into the mitral valve leaflet, are uncommon and associated primarily with hypertrophic cardiomyopathy. Herein, we present a rare case of isolated anomalous papillary muscle insertion into the anterior mitral valve leaflet, causing both dynamic left ventricular outflow tract obstruction and severe mitral regurgitation, in a patient without clear-cut evidence of hypertrophic cardiomyopathy. We discuss potential mechanisms for the hemodynamics observed and underscore the importance of recognizing this clinical entity, as it may alter the surgical treatment.

## 2. Case Presentation

A 59-year-old healthy male sought an outpatient medical evaluation following two episodes of unwitnessed syncope that had occurred two weeks prior. He described the most dramatic event occurring after waking up from sleep in the mid-morning hours to urinate, when he was found by his family lying on the ground unconscious with a minor frontal scalp contusion. He denied any symptoms prior to the fall. His medical history was unremarkable for any atherosclerotic risk factors or rheumatic fever as a child. His vital signs were normal but physical examination was notable for a 2/6 crescendo systolic murmur at the right upper sternal border and a holosystolic murmur at the apex. An electrocardiogram showed normal sinus rhythm with no signs of left ventricular hypertrophy and normal ST segments and T waves. An event monitor did not reveal any evidence of arrhythmia.

**Figure 1:**
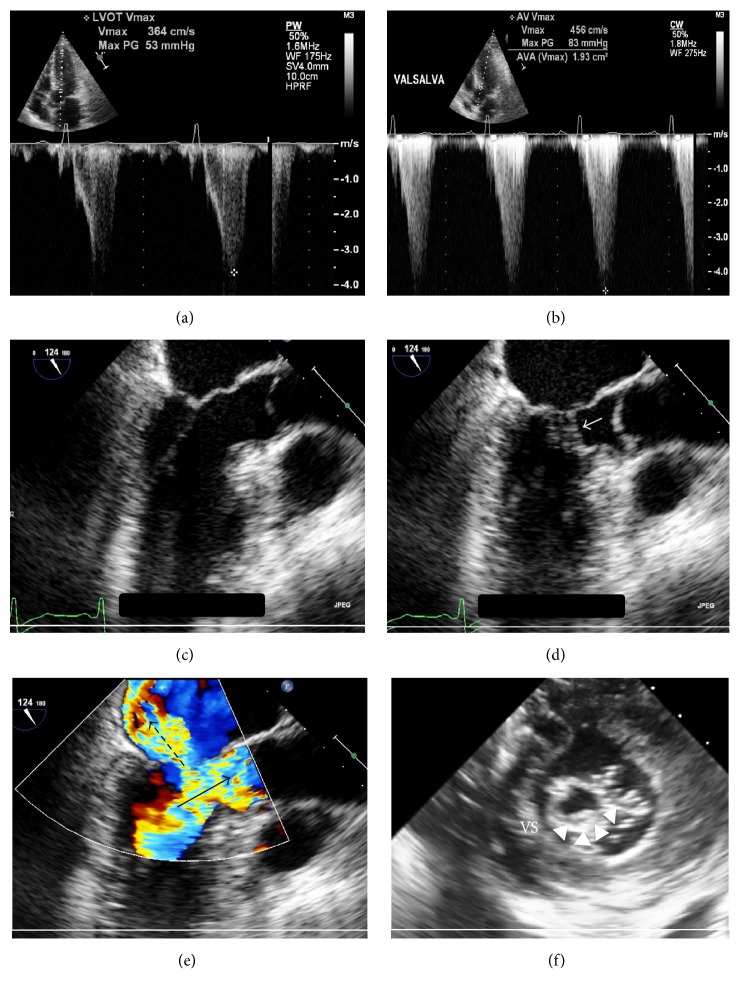
Transthoracic and transesophageal echocardiographic findings. (a) Pulse-wave Doppler at the LVOT showing a peak velocity of 3.94 m/s and gradient 62 mmHg. The waveform contour is dagger-shaped. (b) Strain-phase Valsalva results in an increase in peak velocity to 4.56 m/s with gradient 83 mmHg. (c) Apical view at end-diastole showing no significant LVOT narrowing. The septal wall shows moderately increased thickness measured at 1.4 cm. (d) Apical view during systolic phase showing mitral-septal contact (white arrow) consistent with SAM. (e) Color Doppler showing severe, posteriorly directed mitral regurgitation (dashed arrow) into a moderately enlarged left atrial cavity and significant turbulence across the LVOT (solid arrow). (f) Transgastric short-axis view at the level of the mitral valve during systolic phase, showing a large echodensity attached to lateral scallop of the anterior mitral leaflet (arrowheads).

**Figure 2:**
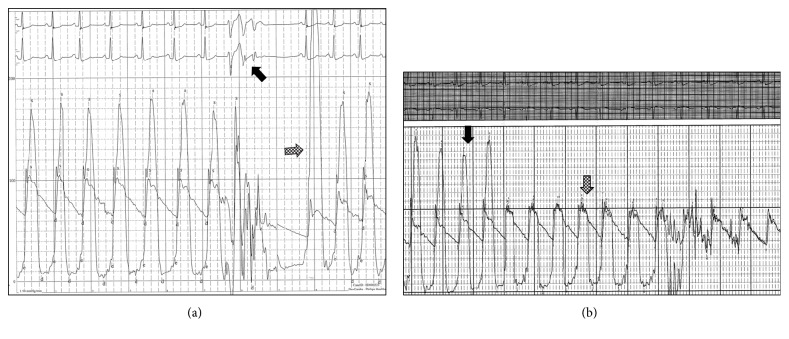
Hemodynamic tracings at cardiac catheterization. (a) Simultaneously measured aortic and LV pressures using a dual lumen pigtail catheter demonstrate an 84 mmHg resting peak-to-peak gradient. The aortic waveform shows a spike-and-dome pattern, and the LV waveform is late-peaking. After premature ventricular contractions are provoked (black arrow), the postextrasystolic beat demonstrates a reduction in aortic pulse pressure with a worsening in peak-to-peak gradient to 160 mmHg (checkered arrow). This finding is consistent with dynamic LVOT obstruction. (b) Pullback tracing with the dual lumen pigtail catheter starting in the mid LV cavity (black arrow) and then being withdrawn to just beneath the aortic valve (checkered arrow) demonstrating normalization of the large gradient. This suggests that the site of obstruction is within the LVOT.

A transthoracic echocardiogram demonstrated normal left ventricular (LV) cavity dimensions and ejection fraction but color Doppler revealed significant turbulence across the LV outflow tract (LVOT). Pulse-wave Doppler of the LVOT revealed a late-peaking “dagger-shaped” systolic jet with a peak velocity of 3.94 m/s and gradient of 62 mmHg that, respectively, increased to 4.56 m/s and 83 mmHg with Valsalva maneuver (Figures 1(a) and 1(b)). There was moderately increased LV wall thickness with the septal wall measuring 1.4 cm. A transesophageal echocardiogram more clearly pointed out the presence of systolic anterior motion (SAM) of the anterior mitral valve leaflet (Figures 1(c) and 1(d)), but there was also a thicker subvalvular structure attached to the ventricular surface of the anterior mitral leaflet which was noted as not typical of chordae tendineae. The anterior mitral valve leaflet appeared redundant, and color Doppler demonstrated a severe, posteriorly directed mitral regurgitant jet into a moderately enlarged left atrial cavity (Figures 1(e) and 1(f)). The trileaflet aortic valve displayed normal mobility. Right and left heart catheterizations showed normal coronary arteries and filling pressures. Using a dual lumen pigtail catheter, simultaneous aortic and LV pressures were recorded showing an 84 mmHg peak-to-peak gradient that increased to 160 mmHg with induction of premature ventricular contractions as well as strain-phase Valsalva (Figure 2(a)). A pullback tracing of the same catheter from the mid LV cavity to just beneath the aortic valve in the LVOT showed normalization of the gradient, corroborating the subvalvular level of obstruction and the lack of aortic valve involvement (Figure 2(b)).

**Figure 3:**
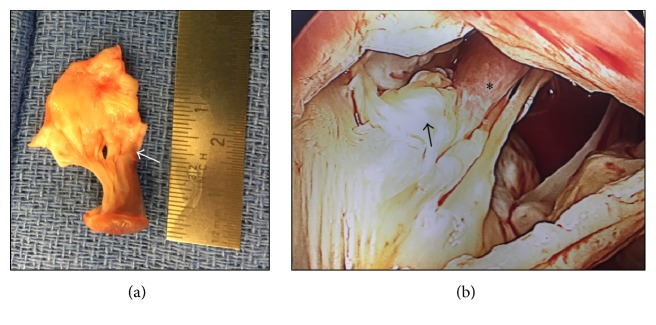
Gross pathology findings. (a) Specimen excised during surgery showing a hypertrophied anterolateral LV papillary muscle attached directly to the anterior mitral leaflet edge with minimal chordae present (white arrow). The anterior leaflet appears myxomatous. (b) In situ view of anomalous anterior papillary muscle (asterisk) insertion directly into anterior mitral leaflet (black arrow) which is more anteriorly displaced and extending across the LVOT.

The final impression following diagnostic testing was that this subject had developed symptomatic, dynamic LVOT obstruction and asymptomatic, chronic severe mitral regurgitation, which were felt to be best treated with cardiothoracic surgery. The operation was performed via a median sternotomy with cardiopulmonary bypass and cardioplegic arrest. After opening the left atrium, the mitral valve leaflets appeared thickened and myxomatous, but notably, the anterolateral papillary muscle was anteriorly displaced and inserted directly into the A1 scallop of the anterior mitral leaflet with minimal chordae tendineae attachments (Figures 3(a) and 3(b)). Furthermore, this displaced papillary muscle seemed to extend directly across the LVOT. Surgical management consisted of excision of the abnormally positioned papillary muscle and A1 portion of the anterior mitral leaflet and replacing this with a bovine pericardial bioprosthesis. The A3 chords of the anterior mitral leaflet and all chords of the posterior leaflet were preserved. A mitral valve repair only strategy was not felt to be a viable option given concerns that this would not provide durable relief from LVOT obstruction. Histologic analysis of the mitral valve tissue revealed nodular myxoid degenerative change, and the papillary muscle was characterized by myofiber hypertrophy. An echocardiogram performed on postoperative day 3 revealed the absence of any significant LVOT turbulence and marked reduction in the transaortic gradient to 14 mmHg. After an uneventful postoperative course, the patient was discharged home alive and well on postoperative day 5. At six-month follow-up, he still reported feeling well with no recurrent syncope.

## 3. Case Discussion/Conclusion

Congenital malformations of the LV papillary muscles, including their direct insertion into various portions of the anterior or posterior mitral valve leaflets, are rare. Our greatest understanding of anomalous papillary muscle insertion (APMI) comes from its identification in subjects with hypertrophic cardiomyopathy (HCM). The largest two case series that included a total of 23 HCM subjects with APMI established its prevalence between 4.5 and 13% [1, 2]. In addition, a few case reports have described the clinical presentation and management of APMI in the HCM population [3–8]. In subjects who do not meet classic criteria for HCM, isolated APMI has been described in only four case reports to date, each describing papillary muscle attachment to either the basal or ventricular surface of the mitral valve leaflets [6, 9–11]. In two other case reports of non-HCM subjects, this aberrancy was shown to be present alongside discrete subaortic stenosis [12, 13].

To our knowledge, this is the first case report to describe APMI in a non-HCM subject leading to two hemodynamic consequences, significant dynamic LVOT obstruction causing syncope and severe mitral valve regurgitation (MR). Dynamic LVOT obstruction that is characteristic of HCM is due to a thickened LV septal wall and anterior mitral valve leaflet SAM that results in mitral-septal contact during systole. Increasingly, primary abnormalities of the mitral valve apparatus in HCM, including abnormal leaflet coaptation, leaflet elongation, chordal slack, and papillary muscle displacement, have been recognized as promoting SAM and generating obstruction [14, 15]. Even in the absence of HCM, SAM has still been shown to occur following treadmill exercise or dobutamine stress [16, 17]. In these settings, intrinsic abnormalities of the mitral apparatus were postulated to be sufficient to cause SAM, independent of significant LV septal wall hypertrophy being present. In the current case, the mechanism of SAM is presumably related to abnormal leaflet coaptation from anterior leaflet restraint induced by the APMI. Furthermore, the anterior displacement of this aberrant papillary muscle may align the anterior mitral leaflet directly within the LV ejection pathway, and so both the papillary muscle and anterior mitral leaflet are swept toward the septum, consistent with previous descriptions [16, 18].

The mechanism of coexistent severe MR appears to be twofold. In HCM subjects, MR is typically posteriorly directed. In the present case, the echocardiographic appearance and histology of the mitral valve leaflets were consistent with myxoid degeneration, and so it is likely that the redundancy of the anterior mitral valve leaflet led to even more compromised coaptation surface. Secondly, the tethering effect of the hypertrophied anterior papillary muscle causing ventricular displacement of the anterior mitral leaflet may have been contributory, as described previously [19]. The role of papillary muscle contraction in exacerbating this tethering effect cannot be discounted. These factors would certainly explain the eccentric nature of the regurgitant jet, which was likely long-standing given the moderate left atrial enlargement observed. Interestingly, the severe mitral regurgitation appears to have been an innocent finding with no clinical symptoms or signs of previous heart failure and normal ventricular filling pressures demonstrated at the time of cardiac catheterization.

For the typical HCM patient with drug-refractory symptoms, surgery with septal myectomy is the first-line therapeutic approach [20, 21]. If mitral valve abnormalities are also present, then mitral valve repair may also be required. For cases of APMI, clinical judgment prevails as there is no standardized surgical approach. A mitral valve-sparing technique described previously involves performing an extensive septal myectomy that extends from the base down to the papillary muscle with a wider trough created toward the apex. By doing so, the mid and apical LV cavity is widened thus relieving the intracavitary obstruction [8, 15]. A different method incorporates mitral valve replacement altogether with concurrent resection of the anomalous papillary muscle, which was the operative technique employed in the current case as well as in two of the previously published case reports of isolated APMI that provided operative details [9, 10]. In all instances, the result was significant reduction in LVOT obstruction. In the first published series of HCM patients with APMI, those who underwent mitral valve replacement achieved a marked reduction in LVOT gradients down to 0–15 mmHg, as opposed to those who only underwent septal myotomy/myectomy who showed persistent 60–70 mmHg gradients and continued symptomatology [1].

The preoperative diagnosis of APMI can be elusive, especially if one does not have knowledge of this congenital variant. In our case, this anomaly was diagnosed by direct intraoperative visualization but only retrospectively fully characterized when reviewing preoperative echocardiographic imaging. This concern has been raised even when examining HCM subjects; as in one series, 90% of necropsy HCM specimens were retrospectively characterized by echocardiography once the diagnosis was elucidated at autopsy [1]. In another series, two-thirds of all subvalvular mitral apparatus anomalies were identified only in the intraoperative setting [2]. This current report of APMI in a non-HCM subject illustrates that invasive hemodynamics generated in the cardiac catheterization laboratory do not reliably differentiate between the various forms of dynamic LVOT obstruction. The diagnosis of APMI relies on clinical knowledge of this entity, optimal echocardiographic delineation of the mitral valve and its subvalvular apparatus, and even cardiac magnetic resonance imaging to understand the mechanisms leading to pathology. Furthermore, a strong index of suspicion is necessary especially if clear-cut HCM is not present.
